# Role of B-Cell Inhibition in Autoimmune Hepatitis: Lessons Learnt From Systemic Lupus Erythematosus

**DOI:** 10.7759/cureus.81108

**Published:** 2025-03-24

**Authors:** Shivangini Duggal, Shrilekha Sairam

**Affiliations:** 1 Internal Medicine, Texas Tech University Health Sciences Center El Paso, El Paso, USA

**Keywords:** autoimmune hepatitis, autoimmune hepatitis (aih), b cell immunotherapy, elevated lft, systemic lupus erythema

## Abstract

Autoimmune hepatitis (AIH) is a chronic liver disease characterized by histological, clinical, and laboratory findings, including elevated liver enzymes, immunoglobulin G (IgG), and autoantibodies. Belimumab, a monoclonal antibody targeting B-lymphocyte stimulator (BLyS), is primarily approved for systemic lupus erythematosus (SLE) but shows promise in improving liver function tests (LFTs) in patients with concurrent AIH and SLE. We present the case of a 24-year-old female diagnosed with AIH and SLE, whose elevated LFTs initially resisted standard azathioprine therapy. Upon switching to mycophenolate and belimumab, a notable reduction in LFTs was observed. This case highlights belimumab’s potential as an adjunct therapy in AIH, especially in patients with overlapping autoimmune conditions, supporting its role in reducing systemic inflammation and autoimmune activity.

## Introduction

Autoimmune hepatitis (AIH) is characterized by chronic, progressive liver inflammation of unknown origin. The diagnosis is based on histological abnormalities, characteristic clinical and laboratory findings (increased aspartate aminotransferase, alanine aminotransferase, and serum IgG concentration), and the presence of one or more characteristic autoantibodies such as anti-smooth muscle antibodies (ASMA) and antinuclear antibodies (ANA); additionally, anti-soluble liver antigen, anti-liver kidney microsomal type 1, anti liver cytosol type 1, and atypical perinuclear antineutrophil cytoplasmic antibodies can be present too [1,2]. Its pathogenesis is strongly linked to human leukocyte antigen (HLA) class II genes, suggesting a key role in CD4+ T-cell responses. Elevated immunoglobulin G (IgG) levels further indicate B cell involvement along with environmental triggers [2].

Belimumab (Benlysta) is a monoclonal antibody that inhibits B-lymphocyte stimulator (BLyS), thereby reducing the survival of B cells, including autoreactive B cells, and decreasing the differentiation of B cells into immunoglobulin-producing plasma cells. This mechanism is particularly relevant in systemic lupus erythematosus (SLE), where B cell hyperactivity plays a central role in disease pathogenesis [3]. Although belimumab is primarily approved for SLE, its role in improving LFTs in patients with concurrent autoimmune hepatitis and SLE is supported by clinical observations. The improvement in LFTs in such patients is likely due to the overall reduction in systemic inflammation and autoantibody production, which positively impacts liver function [4]. Circulating B cell activating factor (BAFF) levels have been found to be elevated in AIH and correlate with markers of liver injury, function, and T cell activation [5]. Studies show that BAFF levels are significantly higher in AIH patients compared to healthy individuals and those with chronic hepatitis C [5]. Additionally, belimumab's ability to reduce glucocorticoid exposure, as demonstrated in clinical trials, may contribute to improved liver function, given the hepatotoxic potential of long-term corticosteroid use [6].

In our patient who presented with AIH, we note a parallel improvement in the patient's liver function tests (LFTs) alongside the resolution of her SLE symptoms with the use of belimumab and mycophenolate.

## Case presentation

A 24-year-old female was referred to our center for evaluation of positive ANA, malar rash, and elevated AST (253 u/L) and ALT (343 u/L). An initial workup revealed a positive ANA titer of 1:320 in a homogenous pattern, negative anti-double-stranded DNA (dsDNA), positive ASMA at 30 u, and elevated immunoglobulin G of 1750 mg/dL. She tested negative for hepatitis (A, B, C), HIV, ceruloplasmin, antimitochondrial antibody, tissue transglutaminase IgA, and alpha-1 antitrypsin. She reported a positive family history of hereditary hemochromatosis and was positive for a C282Y heterozygous mutation for hemochromatosis. However, further iron studies revealed normal iron levels, transferrin, and transferrin saturation (27%). Thus, a probable diagnosis of autoimmune hepatitis was established, based on a revised AIH score of 15. Treatment was initiated with azathioprine (AZA) and hydroxychloroquine, along with prednisone 10 mg (to minimize the risk of corticosteroid-related side effects) for SLE and autoimmune hepatitis.

**Figure 1:**
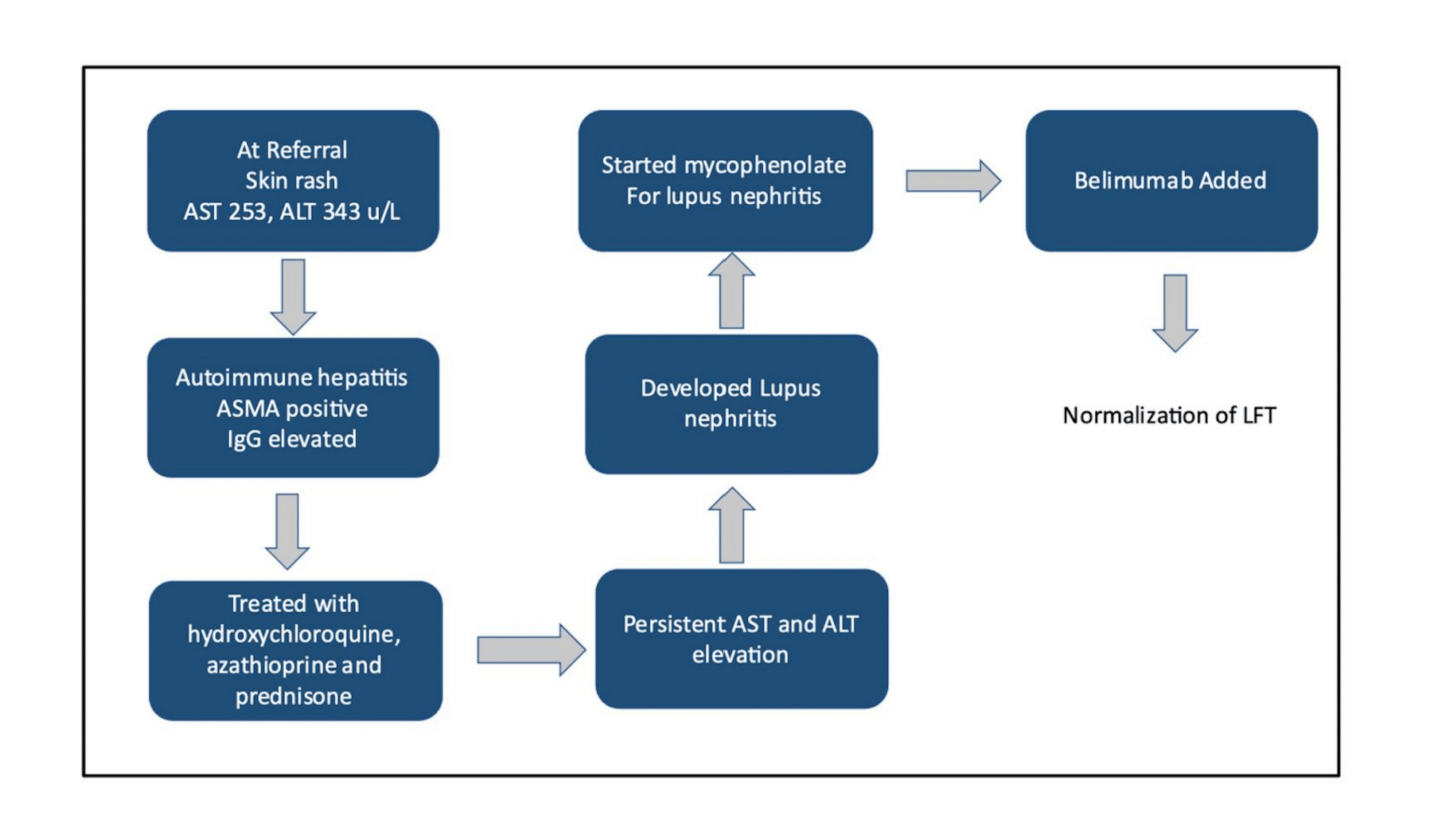
Clinical course of the patient’s disease activity in chronological order. ALT: alanine aminotransferase; AST: aspartate aminotransferase; LFT: liver function tests; ANA: antinuclear antibodies; ASMA: anti-smooth muscle antibodies; IgG: immunoglobulins; SLE: systemic lupus erythematosus; MMF: mycophenolate

During her third visit, her LFTs were noted to be persistently elevated. Thus, the dosage of her azathioprine was increased, and the prednisone dose was maintained at 5 mg. Approximately eight months after her initial presentation, she developed bilateral lower extremity swelling, proteinuria, and hypoalbuminemia, indicating the development of lupus nephritis. She was switched from azathioprine to mycophenolate (MMF) for the management of her lupus nephritis, and her steroid dose was increased. The patient was evaluated as a candidate for belimumab and was started on intravenous belimumab in view of edema and hypoalbuminemia. Normalization of her LFTs was observed with high-dose steroids and remained normal after starting belimumab and taper of steroids (Table 1, Figure 1). The patient continues on mycophenolate and belimumab and is currently doing well.

**Table 1:** Patient’s LFT trends on different medications. ALT: alanine aminotransferase; Alk Phos: alkaline phosphatase; AST: aspartate aminotransferase; LFT: liver function tests

LFT (Normal U/L)	1st Visit Labs at Referral (06/12/2023)	2nd Visit (on Prednisone & Azathioprine) (12/12/2023)	3rd Visit (Increasing Dosage) (1/11/2024)	4th Visit (Continued Increasing Dosage) (2/8/2024)	5th Visit (After Switching to MMF & Adding Belimumab) (9/10/2024)
AST 7-55	253	120	118	115	36
ALT 8-48	343	192	188	161	41
Alk Phos 40-129	77	70	73	96	94

## Discussion

Hepatic involvement in patients with SLE is well documented but considered rare. However, the distinctions between liver involvement due to hepatotoxic drugs, coincident viral hepatitis, non-alcoholic fatty liver disease, or concurrent AIH as a separate condition in SLE patients remain unclear [7]. In our case, the patient had a high score on the revised AIH score, leading to a diagnosis of AIH. Keeping her SLE in mind, she was managed for both at the same time.

Azathioprine, along with steroids, is the standard therapy for the management of AIH; however, multiple studies have shown that only 50% of the patients achieve a long-lasting complete biochemical response (decrease in IgG levels and LFTs) with this therapy [8,9]. Dalekos et al. conducted a study comparing MMF with the standard therapy for AIH and demonstrated that a non-response rate was significantly lower in the MMF group than in the AZA group (7.7 vs 9.3 in MMF vs AZA; p<0.05) and complete biochemical normalization at the end of a follow-up period (96.4 vs 87.2 in MMF vs AZA; p<0.05) was significantly higher in the MMF-treated patients than in the AZA-treated patients [10]. The efficacy of MMF in the treatment of AIH has been proven in multiple retrospective studies and meta-analyses [11-14]. Mycophenolic acid, the active form of MMF, is a selective, potent, reversible, and non-competitive inhibitor of the type II isoform of inosine-5′-monophosphate dehydrogenase. This action results in a targeted immunosuppressive effect with relatively few adverse events, making it ideal for use in transplant patients and individuals with autoimmune diseases. Tolerability to MMF has also been reported in transplant recipients and those with SLE [15]. Our patient demonstrated a similar response to AZA therapy with persistent LFT elevation. Notably, while MMF demonstrates high efficacy in treatment-naïve AIH patients [11], its response rates as a second-line therapy in difficult-to-treat cases range from 32% to 82% across different cohorts. It has been observed that it tends to be more effective in patients who discontinue AZA due to intolerance or adverse effects [11,14].

Psenak et al. described a case of a 60-year-old male with cutaneous SLE, who presented to them with elevated hepatitis. They administered MMF and belimumab, achieving resolution of the LFTs soon after administration; similar findings have been demonstrated in our case [16]. Currently, belimumab is primarily indicated as an add-on therapy for adults with active, autoantibody-positive SLE who have significant disease activity in the skin and/or musculoskeletal systems that persists despite optimized standard immunosuppressive treatment. Kolev et al. recently investigated the treatment response of six patients with AIH and primary biliary cholangitis (PBC) with or without concomitant Sjogren’s syndrome discovering that all patients with AIH and Sjogren’s syndrome had excellent disease control and symptom improvement with belimumab [17].

Belimumab is a human monoclonal antibody that inhibits soluble B-cell activating factor (BAFF), also known as B-lymphocyte stimulator (BLyS). BAFF or BLyS is a type II transmembrane protein expressed on myeloid cells and can also exist as a soluble molecule after being cleaved by proteases [16]. It belongs to the TNF family and plays a crucial role in B cell maturation, proliferation, and survival through its interaction with three receptors: BAFF-R (BAFF receptor), TACI (transmembrane activator and calcium modulator and cyclophilin ligand (CAML) interactor), and BCMA (B cell maturation antigen). BAFF enhances the survival of transitional B cells, promotes immunoglobulin production, and upregulates anti-apoptotic genes such as Bcl-2 and Bcl-xL [18]. Overexpression of BAFF can lead to autoimmune manifestations, such as hypergammaglobulinemia and autoantibody production. It also influences T cell activation and survival through BAFF-R and can modulate immune responses by promoting IL-10 production, creating a feedback loop that affects both inflammatory and immunosuppressive pathways. In autoimmune diseases such as SLE, elevated BAFF levels contribute to the disrupted balance of immune regulation, enhancing both B- and T-cell activity [19].

Circulating BAFF levels have been found to be elevated in AIH and correlate with markers of liver injury, function, and T-cell activation [20]. These levels are strongly correlated with serum AST, ALT, bilirubin, and soluble CD30, a marker of T-cell activation [21]. Interestingly, BAFF levels improve markedly after two weeks of glucocorticoid therapy, paralleling reductions in ALT. The correlation between BAFF and soluble CD30 suggests a T-cell-mediated pathogenic pathway in AIH. Additionally, BAFF levels correlate with histological severity, showing higher levels in patients with more advanced liver inflammation and fibrosis. This association between BAFF and active liver inflammation is also evident during AIH relapse, highlighting its potential role in disease progression [21,22]. A multicenter phase II/III trial (NCT03217422) is underway to estimate the efficacy of ianalumab (another BAFF receptor inhibitor) in patients with AIH non-responding to standard treatment.

## Conclusions

In conclusion, belimumab shows promise in managing probable AIH as a steroid-sparing agent, particularly in patients with concurrent SLE or Sjogren. Its mechanism-reducing autoreactive B cells and decreasing systemic inflammation support improved liver function, as evidenced in this case report, where belimumab led to normalization of liver function tests, taper of steroids, and resolution of SLE symptoms. The correlation of elevated BAFF levels with liver injury and inflammation in AIH further underscores its role in disease pathogenesis.
